# Production of Hybrid Chimeric PVX Particles Using a Combination of TMV and PVX-Based Expression Vectors

**DOI:** 10.3389/fbioe.2015.00189

**Published:** 2015-11-20

**Authors:** Christina Dickmeis, Mareike Michaela Antonia Honickel, Rainer Fischer, Ulrich Commandeur

**Affiliations:** ^1^Institute for Molecular Biotechnology, RWTH Aachen University, Aachen, Germany; ^2^Fraunhofer Institute for Molecular Biology and Applied Ecology, Aachen, Germany

**Keywords:** *potato virus X*, *tobacco mosaic virus*, hybrid chimeric particles, viral vectors, coexpression, fluorescent proteins, nanoparticles

## Abstract

We have generated hybrid chimeric *potato virus X* (PVX) particles by coexpression of different PVX coat protein fusions utilizing *tobacco mosaic virus* (TMV) and PVX-based expression vectors. Coinfection was achieved with a modified PVX overcoat vector displaying a fluorescent protein and a TMV vector expressing another PVX fluorescent overcoat fusion protein. Coexpression of the PVX-CP fusions in the same cells was confirmed by epifluorescence microscopy. Labeling with specific antibodies and transmission electron microscopy revealed chimeric particles displaying green fluorescent protein and mCherry on the surface. These data were corroborated by bimolecular fluorescence complementation. We used split-mCherry fragments as PVX coat fusions and confirmed an interaction between the split-mCherry fragments in coinfected cells. The presence of assembled split-mCherry on the surface confirmed the hybrid character of the chimeric particles.

## Introduction

Plant virus particles offer an excellent tool for pharmaceutical and nanotechnological applications. They can consist of multiple identical copies of one or two coat protein (CP) subunits and can be easily modified by genetic engineering or chemical conjugation (Meunier et al., 2004; Chatterji et al., 2005; Steinmetz, 2010; Rego et al., 2013; Lee et al., 2014). Plant virus capsids offer suitable building blocks for areas of research and application as diverse as electronic devices, composite materials, and vaccine production. There is a growing demand for inexpensive vaccines that can be distributed and stored without maintaining the cold chain, particularly in developing countries which lack an appropriate infrastructure (Ma et al., 2005b). This exacerbates even simple infectious diseases and ultimately leads to a high child mortality rate (Hefferon, 2013). Although plant virus particles might also need cold storage after purification, the lifetime and stability in plant material is better compared to other vaccine production systems.

Plants are considered a promising alternative production system for pharmaceuticals and have been studied extensively over the past decades (Fischer et al., 2012; Lico et al., 2012; Melnik and Stoger, 2013). Plant virus particles or CPs are ideal for the presentation of epitopes (Porta and Lomonossoff, 1998; Yusibov et al., 2006; Morgenfeld et al., 2009; Rosenthal et al., 2014). They can be produced in large amounts in plants as bioreactors, with inexpensive growth conditions and a minimal risk of contamination with animal pathogens (Twyman et al., 2003; Ma et al., 2005a). Plant virus particles and CPs as carrier molecules enhance the immunogenicity of peptides by presenting them robustly to the immune system (Lomonossoff and Evans, 2014). When the target epitope is inserted at an appropriate site on the selected plant viral CP, they can assemble to intact particles displaying the foreign peptide on the outer surface (Johnson et al., 1997). Immune responses against single pathogen epitopes are in most cases not sufficient to provide protection against an infection. Often different genotypes or subtypes of pathogens are found, e.g., the mammalian virus *Hepatitis C virus* (HCV) (Simmonds et al., 2005). This makes a vaccine development difficult, which is further complicated by the degree of heterogeneity in infected individuals due to the pathogen mutation rate (Hayashi et al., 1999). The presentation of several different epitopes from the same pathogen or from several different pathogens is ideal for the construction of efficient vaccines (Sette et al., 2001; Awram et al., 2002).

Several plant viruses have been developed as epitope presentation systems. The most widely used are *cowpea mosaic virus* (CPMV) (Brennan et al., 2001; Gonzalez et al., 2009; Sainsbury et al., 2010) and *tobacco mosaic virus* (TMV) (Sugiyama et al., 1995; Porta and Lomonossoff, 1998). Another promising plant virus for epitope presentation is *potato virus X* (PVX), the type member of the genus *Potexvirus* in the virus family *Flexiviridae* (Adams et al., 2004). *Solanaceae* (e.g., potato, tomato, and tobacco) are infected by PVX and the virus is transmitted mechanically from plant to plant (Koenig, 1971). PVX has a +ssRNA genome 6.4 kb in length, with a 5'-methylguanosine cap, a polyadenylated 3' tail, and five ORFs (Koonin, 1991; Kim and Hemenway, 1997). The RNA-dependent RNA polymerase (RdRp) is the first ORF and is the only virus protein needed for viral RNA synthesis and replication (Draghici et al., 2009). The next three ORFs overlap, and encode the p25, p12, and p8 triple gene block (TGB) proteins necessary for the cell-to-cell transport (Beck et al., 1991; Angell and Baulcombe, 1997; Verchot et al., 1998). The last ORF at the 3' end encodes the CP. Approximately 1270 CP subunits assemble with the plus-strand RNA into the viral particle (Tollin and Wilson, 1988). Furthermore, the CP is also important for cell-to-cell movement and long distance transport through the plant (Chapman et al., 1992; Baulcombe et al., 1995; Cruz et al., 1998). The virus particles have a size of 515 nm × 13.5 nm and are flexuous rods (Tollin and Wilson, 1988; Atabekov et al., 2007). Flexuous particles have, in general, the advantage of no intrinsic size limitations or packaging constraints and potentially many copies of the target peptide can be presented on the surface (Uhde et al., 2005). The N-terminal portion of the PVX CP is exposed on the virion surface (Koenig and Torrance, 1986; Baratova et al., 1992), making N-terminal fusions to the CP the ideal strategy for epitope presentation. Several epitopes had been presented as PVX CP fusions, including the 2F5 epitope of the *human immunodeficiency virus* (HIV) (Marusic et al., 2001), an epitope of the *Influenza A virus* nucleoprotein (Lico et al., 2009), and the R9 peptide of HCV (Uhde-Holzem et al., 2010). In all these cases, robust immune responses were achieved in animal models.

Limiting factors for peptide fusions to the PVX CP is the size and the isoelectric point (pI) of the target epitope. The inserted sequences can impair virus assembly and systemic infection (Uhde-Holzem et al., 2007). Thus far, the maximum epitope sequence that has been displayed as a direct CP fusion on the particle surface was 14 residues in length (Uhde-Holzem et al., 2010). For the display of larger sequences, the 2A sequence from *foot and mouth disease virus* (FMDV) is inserted between the peptide and the CP. The 2A sequence confers a cotranslational ribosomal skip, leading to a production of non-fused CP and CP fusion product in a certain ratio depending on the amino acid sequence (Donnelly et al., 2001a,b). In this way, complete proteins can be displayed on the particle surface, creating the so-called overcoat (Cruz et al., 1996), which allows PVX particles to be used, for example, as optical probes for imaging in plant cells (Linnik et al., 2013). The stability and functionality of these particles were also confirmed in mammalian cells and tissues (Shukla et al., 2014).

In this study, we created a production system for hybrid chimeric PVX particles displaying different polypeptides as CP fusions. For this, we used combinations of PVX and TMV expression vectors, each expressing different PVX CP fusions. The coinfection capacity of PVX and TMV vectors has been described earlier and is, for example, used for the production of recombinant antibodies (Giritch et al., 2006), confirming the coexpression of different transgenes in the same cells. To prove the principle of the assembly of hybrid chimeric PVX virions, we chose the green fluorescent protein (GFP) and the red fluorescent protein mCherry, as well as a bimolecular fluorescence complementation (BiFC) system with split-mCherry as CP fusion proteins.

## Materials and Methods

### Plasmid Constructs

The expression constructs containing the GFP and mCherry CP fusions were assembled using PCR to add *Pac*I and *Not*I restriction sites. The PVX expression vectors pTCXI (GFP-2A-CP) and pTCXIIc (mCherry-2A-CP) were used as templates (Shukla et al., 2014). Forward primers *Pac*I-GFP (5′-AGT TAA TTA ATG AGT AAA GGA GAA G-3′) and *Pac*I-mCherry (5′-AGT TAA TTA ATG GTG AGC AAG GGC G-3′) with reverse primer CP-*Not*I (5′-TTG CGG CCG CTT ATG GTG GTG GTA G-3′) were used. The resulting PCR products and the target TMV vector pJL36 [kindly provided by Lindbo (2007)] were digested with *Pac*I and *Not*I (NEB). The final constructs were prepared by joining the fragments with T4-DNA ligase (Promega) overnight at 16°C. The ligation products were introduced into *Escherichia coli* DH5α and colonies were selected on LB plates supplemented with 50 mg/ml kanamycin overnight at 37°C. Plasmid DNA was isolated from overnight cultures and sequenced. The resulting plasmids were called pTMV-GFP-2A-CP_PVX_ and pTMV-mCherry-2A-CP_PVX_. The split-mCherry sequence was constructed essentially as described by Fan et al. (2008), with the mCherry split-site at residues 159/160. Either the C-terminal (*Nhe*I-C-mC: 5′-ACC AGC TAG CGC TAC CGG TCG CCA CCA TGG GCG CCC TGA AGG GCG AGA TC-3′ and mCherry-*Bsp*EI: -5′-TCC GGA CTT GTA CAG CTC GTC CAT-3′) or N-terminal (N-mC-*Bsp*EI: 5′-CTT AAG AAG GTC AAA ATT TCT AGA TCC GGA GTC CTC GGG GTA CAT CCG CT-3′ and TGB-fw: 5′-AAG GGC CAT TGC CGA TCT CAA GC-3′) part of mCherry was amplified by PCR, adding the restriction sites *Nhe*I and *Bsp*EI for cloning in the PVX vector. After construction of the PVX vectors with the split-mCherry components (pPVX-N-mC-2A-CP and pPVX-C-mC-2A-CP), the sequence with the CP fusion construct was amplified by PCR adding the restriction sites *Pac*I (*Pac*I-N-mC: 5′-AGT TAA TTA ATG GTG AGC AAG GGC G-3′ or *Pac*I-C-mC: 5′-CGT GTT CTT GTC ATT AAT TAA TGG GCG CCC TGA AGG GCG AGA TC-3′) and *Not*I (CP-*Not*I) for cloning into the TMV vector, resulting in expression vectors pTMV-N-mC-2A-CP_PVX_ and pTMV-C-mC-2A-CP_PVX_.

### Plant Inoculation and Maintenance

For inoculation with PVX vectors, 4-week-old *Nicotiana benthamiana* plants were dusted with Celite 545 (3–4 leaves per plant) and then rubbed with 10 μg plasmid DNA per leaf. PVX vectors were introduced into *Agrobacterium tumefaciens* strain GV3101:pMP90RK and TMV vectors were introduced into *A. tumefaciens* strain GV2260. The bacteria were cultivated at 26°C in YEB medium (0.5% beef extract, 0.1% yeast extract, 0.5% peptone, 0.5% sucrose, and 2 mM MgSO_4_) supplemented with 100 mg/l carbenicillin, 50 mg/l rifampicin, and 25 mg/l kanamycin. The cultures were pre-induced with 10 mM MES (pH 5.6), 10 mM glucose, and 20 μM acetosyringone after 24 h of growth. After 48 h, the cultures were diluted to OD_600nm_ = 0.5 for TMV vectors and OD_600nm_ = 1 for PVX vectors with 2× infiltration medium [100 g/l sucrose, 3.6 g/l glucose, 8.6 g/l Murashige and Skoog (MS) salts, pH = 5.6] supplemented with 200 μM acetosyringone and incubated for 30 min at room temperature. Four-week-old *N. benthamiana* plants were then inoculated by syringe without a needle and incubated at 26°C for 12 h in the dark at 20°C for 12 h in a phytochamber with constant light (25,000–30,000 lux).

### Protein Isolation and Analysis

Total soluble protein was isolated from harvested leaves by homogenizing leaf tissue in two volumes of phosphate-buffered saline (PBS). Insoluble debris was removed by centrifugation (13,000 rpm, 10 min, 4°C) and protein samples were resolved by SDS-PAGE (Laemmli, 1970) after mixing plant sap with 5× reducing buffer (62.5 mM Tris–HCl pH 6.8, 30% glycerol, 4% SDS, 10% 2-mercaptoethanol, and 0.05% bromophenol blue). The samples were either visualized directly in the gel to confirm the presence of the fluorescent proteins or they were boiled for 5–10 min for western blotting. Samples were separated on 12% polyacrylamide-SDS gels and then either stained with Coomassie Brilliant Blue or blotted onto a nitrocellulose membrane (HybondC, Amersham) for western blot analysis. The membranes were blocked for 1 h with 5% skimmed milk in PBS and incubated with a polyclonal antibody against PVX CP (DSMZ, Braunschweig, Germany), TMV CP (Bioreba, Switzerland), DsRed, or GFP (GeneTex, Irvine, CA, USA) for at least 2 h at room temperature. The DsRed antibody recognizes mCherry because the latter is a monomeric form directly derived from DsRed. A monoclonal alkaline phosphatase-conjugated goat anti-rabbit antibody (GAR^AP^) was used as the secondary antibody (Dianova, Hamburg, Germany) and the signal was visualized with nitroblue tetrazolium chloride/5-bromo-4-chloro-3-indolyphosphate p-toluidine salt (NBT/BCIP).

### Detection of Fluorescence

The plants were monitored daily and analyzed for GFP fluorescence using a handheld UV lamp (7000 μW, Novodirect, Kehl/Rhein, Germany) and for mCherry fluorescence with green light (KL2500 LCD, Schott AG, Mainz, Germany) and a red filter. Pictures were taken with a Nikon Coolpix 5400 camera (Nikon, Düsseldorf, Germany). For microscopic analysis, plant parts were analyzed with a Biorevo BZ-9000 fluorescence microscope (Keyence, Neu-Isenburg, Germany).

### Particle Purification

Plant material was harvested 14–21 dpi depending on the infection status, and 50 g of plant material was used for virus purification with a modified protocol from CIP (International Potato Center, Lima, Peru), as previously described (Uhde-Holzem et al., 2010). PEG precipitation was carried out as described in the original protocol, but the centrifugation on a sucrose cushion was skipped because we lost too many particles during this step. Therefore, the centrifugation of the pooled fractions of the sucrose gradient continued for at least 3 h. The virus concentration was determined at OD_260nm_ and the extinction coefficient of 3.0 for TMV because the TMV concentration in the mixtures tended to be greater.

### Transmission Electron Microscopy

The particles were either directly adsorped to grids for transmission electron microscopy (TEM), or processed for immunosorbent transmission electron microscopy (ISEM) as followed. For the adsorption grids, 20 μg of purified particles was incubated on Pioloform-coated 400-mesh nickel grids for 20 min. For ISEM, the grids were coated with the first antibody either α-PVX, α-DsRed or α-GFP antibodies diluted 1:10 in PBS for 20 min. Unbound antibodies were washed off with PBST [PBS supplemented with 0.2% (w/v) Tween 20] and the grids were blocked with 0.5% (v/v) bovine serum albumin (BSA) in PBS for 15 min. Subsequently, the grids were incubated with 20 μg of the particle preparation for 20 min, followed by washing with PBS. Captured PVX particles were analyzed for fluorescent proteins on the surface by immunogold staining. Therefore, the preparations were incubated for 2 h with the α-PVX, α-mCherry, or α-GFP antibodies diluted 1:100 in PBS. Primary antibodies were detected by overnight incubation with goat anti-rabbit secondary antibodies labeled with 15-nm gold particles diluted 1:50 in PBS, recognizing the bound specific antibodies. Afterward, the grids were washed thoroughly with PBS and then with distilled water and counterstained with 1% (w/v) uranyl acetate (pH 4.3) before analysis with a Zeiss EM 10 TEM (Oberkochen, Germany).

## Results

### Coexpression of PVX-GFP-2A-CP and TMV-mCherry-2A-CP_PVX_

**Figure 1:**
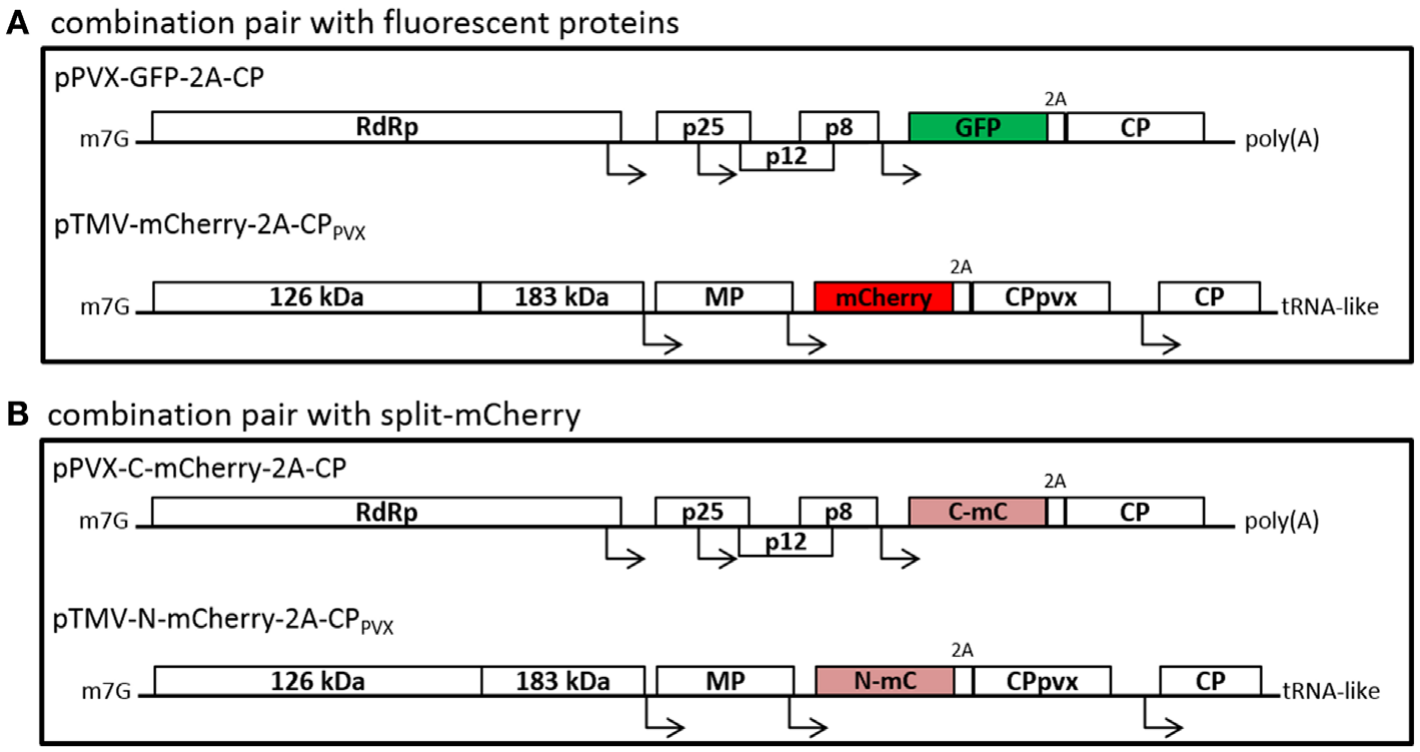
**Schematic overview of the used plant viral vectors with fluorescent proteins (A) or split-mCherry (B)**. **(A)** PVX-based vectors with fluorescent protein fusions with the help of the 2A sequence of the FDMV are used, as for example the GFP-2A-fusion is shown. The PVX CP fusion was introduced into TMV-based expression vectors, either with mCherry or with GFP. **(B)** As second option, split-mCherry was used instead of fluorescent proteins. Therefore, either the N- or C-terminal part of mCherry was cloned into the viral vectors. RdRp, RNA-dependent RNA polymerase; p25, p12, and p8, triple gene block proteins; CP, coat protein; 126 and 183 kDa, replicase complex of TMV; MP, movement protein; m7G, methyl guanidine cap; poly(A), polyadenylated tail; tRNA-like, tRNA-like structure of TMV.

We constructed TMV-based vectors expressing the PVX CP joined to fluorescent proteins mCherry or GFP via a FDMV 2A peptide (Figure 1). We used the mCherry-2A-CP_PVX_ and GFP-2A-CP_PVX_ fusions from PVX vectors, which incorporate the mCherry/GFP-2A-CP_PVX_ fusion along with the wt CP_PVX_ into the particle, as previously reported (Shukla et al., 2014). These CP_PVX_ fusion sequences were cloned into the expression cassette of the TMV-based vector pJL36 (Lindbo, 2007). The resulting vectors (pTMV-mCherry-2A-CP_PVX_ and pTMV-GFP-2A-CP_PVX_) express the PVX CP fusions under the control of an additional heterologous subgenomic promoter-like sequence. Viral vectors were inoculated into plants and coexpressed with a PVX vector expressing the corresponding other fluorescent PVX CP fusion protein.

**Figure 2:**
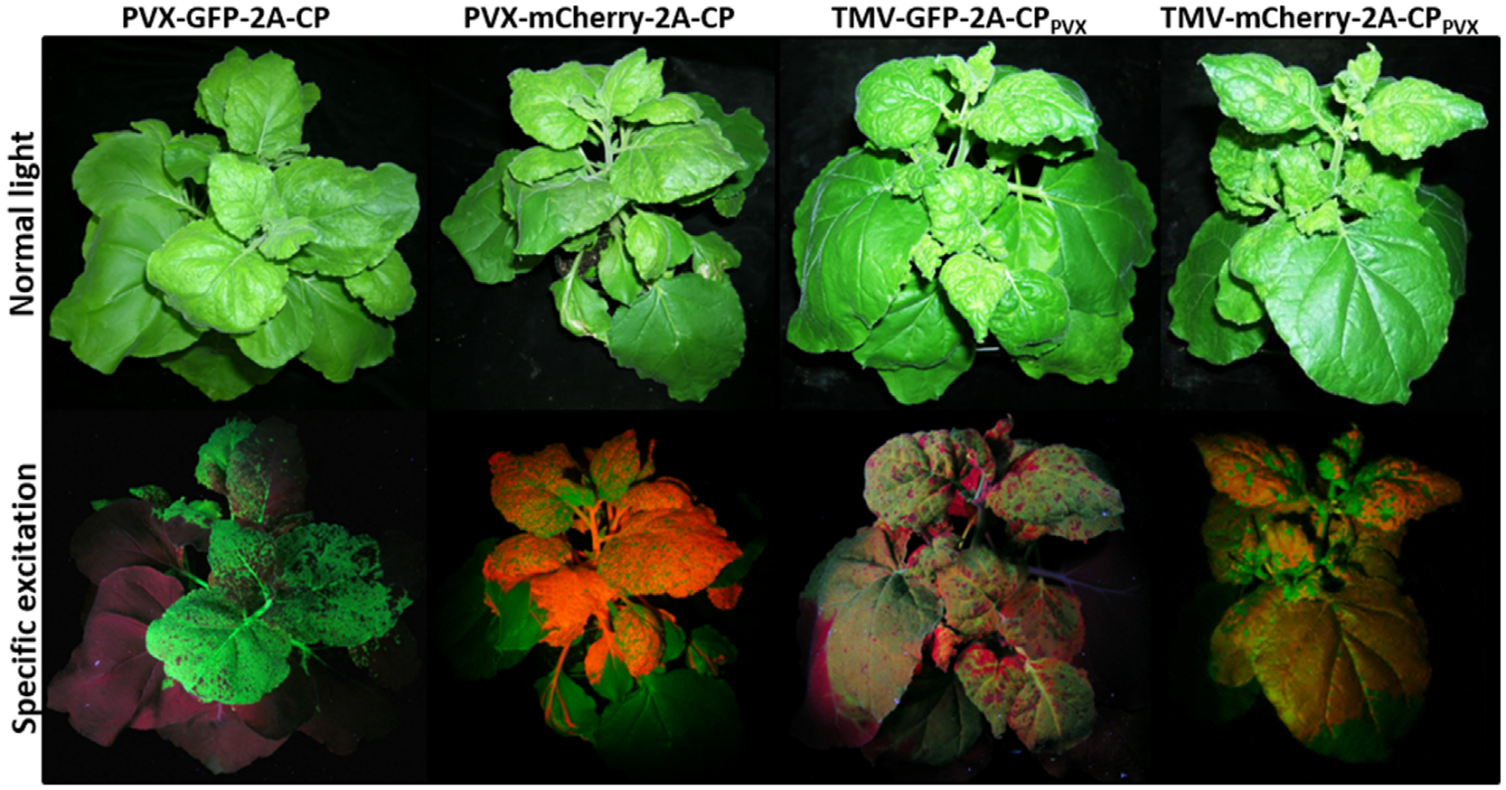
***Nicotiana benthamiana* plants at 12 dpi expressing 2A-CP_PVX_ fusions with fluorescent proteins in PVX or TMV vectors**. The plants are shown under normal light and with specific excitation of the fluorescent proteins. For GFP, the plants are shown under UV light, and for mCherry, they are shown under green light with a red filter.

**Figure 3:**
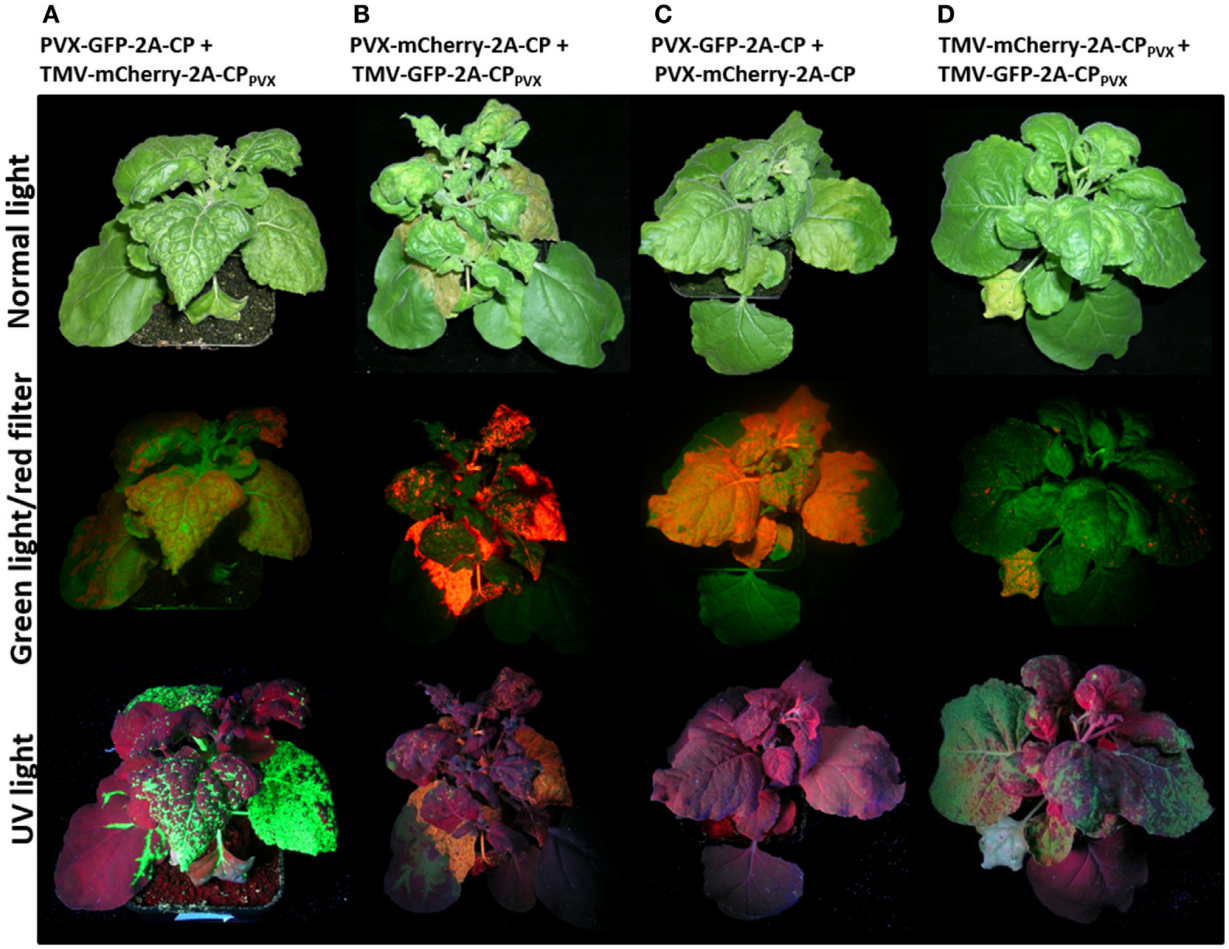
***Nicotiana benthamiana* plants coinfected with PVX and TMV vectors expressing CP fusions of PVX at 12 dpi displayed as whole plant**. Plants were coinfected with **(A)** PVX-GFP-2A-CP and TMV-mCherry-2A-CP_PVX_, **(B)** PVX-mCherry-2A-CP and TMV-GFP-2A-CP_PVX_, **(C)** PVX-GFP-2A-CP and PVX-mCherry-2A-CP, and **(D)** TMV-mCherry-2A-CP_PVX_ and TMV-GFP-2A-CP_PVX_. The plants are shown under normal light and with excitation of the fluorescent proteins (UV light for GFP and green light with a red filter for mCherry).

Plants infected with the individual viral vectors showed systemic infection and overall strong GFP or mCherry expression in the infected leaves, as well as expression of the PVX and TMV CP (Figure 2; Figure S1 in Supplementary Material). The fluorescence intensity was lower with the TMV vectors than the PVX vectors. Coinfections with two vectors based on the same virus (e.g., PVX-GFP-2A-CP and PVX-mCherry-2A-CP) led to the spatial separation of the viruses as they spread through the host plant (Figures 3C,D), and in most cases, one vector became dominant. However, in coinfections with PVX and TMV vectors expressing different CP_PVX_ fusions, we observed the coexpression of the different fluorescent proteins in the same parts of the plant (Figures 3A,B). Furthermore, the expression of the PVX-based fluorescent protein was enhanced in coinfections with TMV.

**Figure 4:**
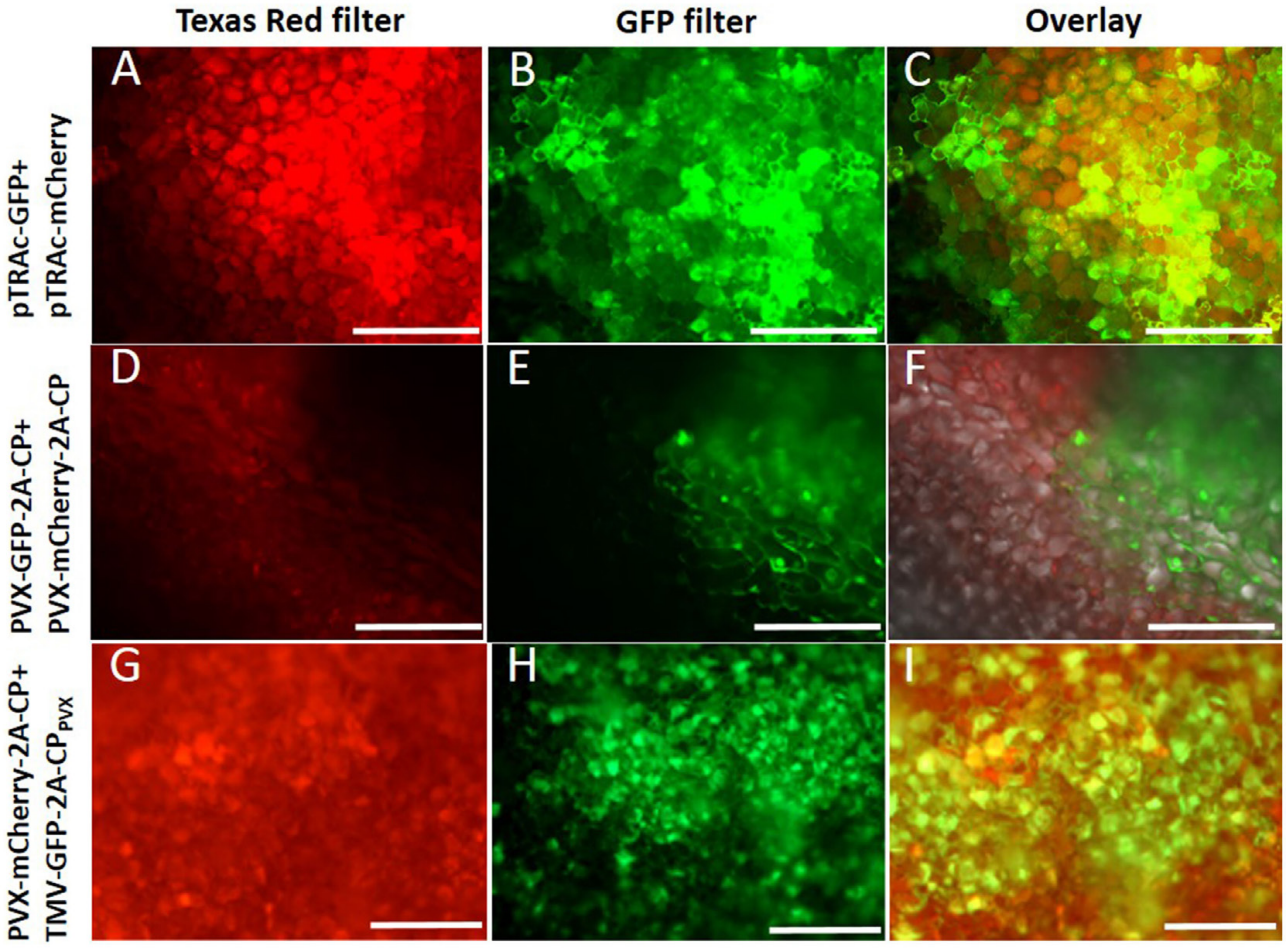
**Plant cells were analyzed by fluorescence microscopy with a 10× magnification**. **(A–C)** Coexpression of GFP and mCherry with pTRAc vectors (positive control), **(D–F)** coinfection with PVX-GFP-2A-CP and PVX-mCherry-CP, **(G–I)** coinfection of PVX-mCherry-2A-CP and TMV-GFP-2A-CP_PVX_; **(A,D,G)** cells shown with excitation of mCherry (Texas red filter), **(B,E,H)** cells shown with GFP excitation, **(C,F,I)** overlay of the pictures for the shown coexpression.

The coexpression of both fluorescent proteins in the same plant cells was confirmed by epifluorescence microscopy (Figure 4). Fluorescent proteins were also transiently expressed as a control by agroinfiltration, using the pTRA vector (Maclean et al., 2007). Plant cells coinfiltrated with both vectors showed simultaneous GFP and mCherry expression, which was confirmed by the yellow color in the overlay picture (Figures 4A–C). In plants coinfected with two PVX vectors expressing different CP fusions (Figures 4D–F) or TMV vectors (data not shown), no coexpression was observed. Vectors based on the same virus backbones excluded each other, and sharp boundaries for GFP or mCherry expression were noted. In contrast, a combination of TMV- and PVX-based vectors coinfected the same cells, both expressing a PVX CP fusion protein (Figures 4G–I).

SDS-PAGE analyses allowed direct visualization of the fluorescent proteins in gels, and the confirmation of CP expression in western blots (Figure S1 in Supplementary Material and data not shown). These experiments confirmed the coexpression of different CP_PVX_ fusions in the same leaves and the same cells following coinfection with PVX and TMV vectors, but did not reveal the PVX particle composition.

### Particle Analysis Presenting Full-Size Fluorescent Proteins

**Figure 5:**
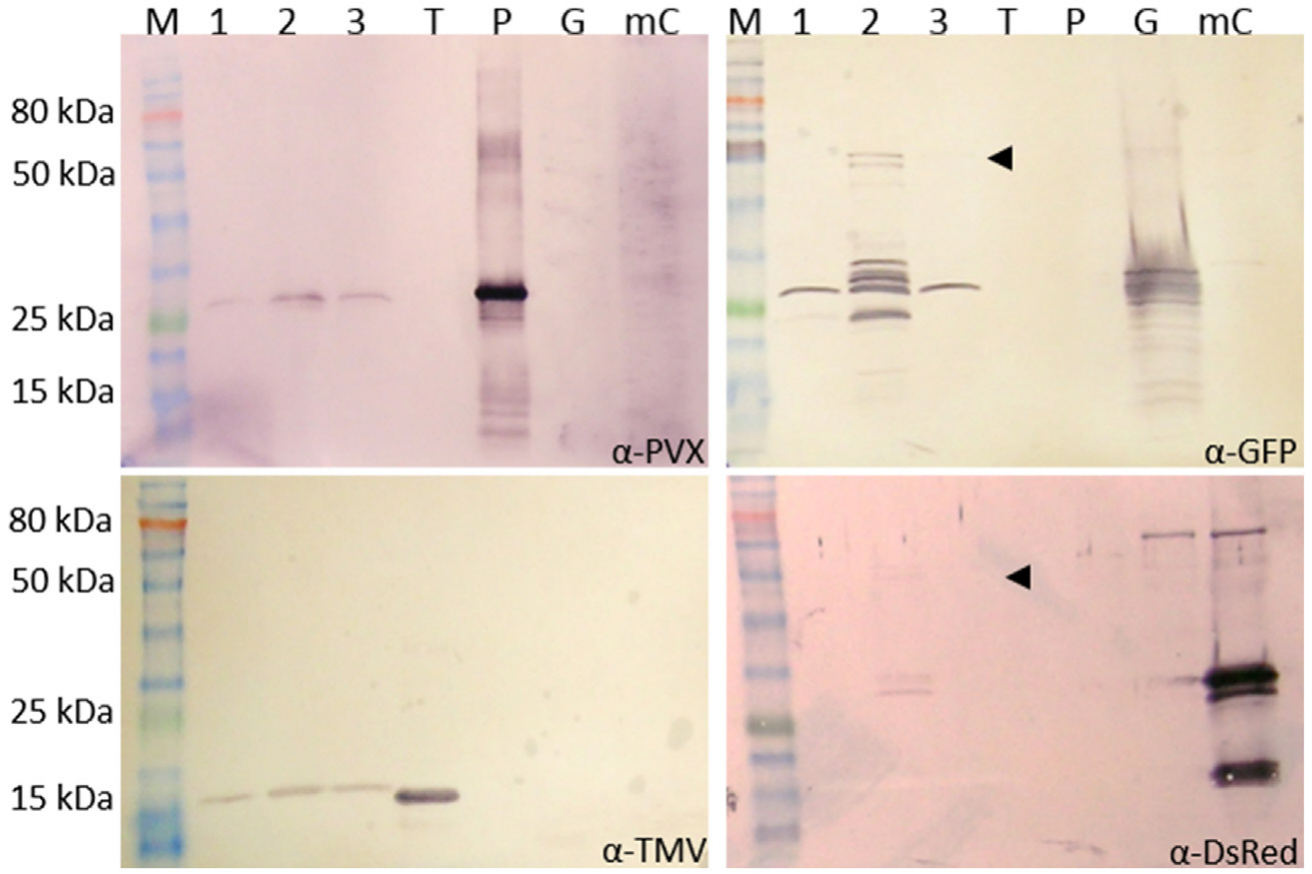
**Analysis of purified PVX and TMV particles with GFP-2A-CP_PVX_ and mCherry-2A-CP_PVX_ fusions**. The particles were purified from a PVX-GFP-2A-CP plus TMV-mCherry-2A-CP_PVX_ coinfection and the preparation was analyzed by western blot to detect CP_PVX_ (α-PVX), CP_TMV_ (α-TMV), GFP (α-GFP), and mCherry (α-DsRed) using GAR^AP^ as the secondary antibody. M: P7711S protein ladder (NEB), 1–3: different pooled fractions of the preparation, T: purified TMV particles (0.5 μg), P: purified PVX particles (0.5 μg), G: GFP control (plant sap expressing GFP from a pTRAc vector), mC: mCherry control (plant sap expressing mCherry from a pTRAc vector). For PVX, GFP, and mCherry, 3 μg of the particle mixture was separated by SDS-PAGE. Only 1 μg was used for TMV analysis. The pooled fraction 2 was analyzed in TEM (Figure 6). The same amount of purified particles (10 μg) was used for the grids.

Virus particles were purified from the coinfected plant material and the particle structure was analyzed to find out whether both fluorescent proteins can be incorporated into the same particles. Following purification, the particle preparations were analyzed by western blot and TEM, using specific antibodies against mCherry and GFP. TMV and PVX particles co-accumulated during purification because the process does not separate them. SDS-PAGE revealed the presence of excess TMV particles compared to PVX, with an approximate ratio of 3:1 (Figure S1 in Supplementary Material; Figure 5). Consequently, 1 μg of the particle preparation was used for TMV analysis, and 3 μg was used for PVX, GFP, and mCherry analysis.

Western blots confirmed the presence of CP_TMV_ (17.5 kDa) and CP_PVX_ (25 kDa) in the preparation. GFP (25 kDa) was detected in all pooled fractions, but only pooled fraction 2 contained the GFP-CP_PVX_ fusion product (Figure 5, indicated by the arrowhead, 54 kDa). Small amounts of the mCherry fusion product were also detected in this fraction using the DsRed antibody (54 kDa). This confirmed the presence of PVX particles with either GFP or mCherry overcoat fusions (Figure 5).

To determine whether both fluorescent protein overcoat structures are found on the same particles, the particles were either directly absorbed to grids for TEM analysis or captured with specific antibodies recognizing the fluorescent proteins or PVX. Control PVX and TMV particles were not captured or labeled with the GFP-specific or DsRed-specific antibodies, confirming the specific binding of these antibodies to the fluorescent proteins (Figure S2 in Supplementary Material). The TMV particles were generally not captured or labeled by the PVX-specific antibody, but a few undecorated particles were found on all grids with TMV. Cross-reactivity of the antibodies against the other fluorescent protein was excluded by purifying either the PVX-GFP-2A-CP or PVX-mCherry-2A-CP particles. Particles displaying a GFP overcoat were only captured and decorated by α-GFP, and mCherry overcoat structures were only labeled and captured by α-DsRed (Figure S2 in Supplementary Material). In the virus preparation from plants coinfected with PVX-GFP-2A-CP and TMV-mCherry-2A-CP_PVX_, a mixture of TMV and PVX particles was observed, because the PVX purification process cannot separate these viruses. The TMV particles are shorter (~300 nm) and have a rigid structure, whereas the PVX particles are >500 nm in length and have a flexible structure, thus allowing the particles to be visually distinguished on the grids. Particles from the coexpression were captured and labeled with both the GFP-specific and DsRed-specific antibodies.

**Figure 6:**
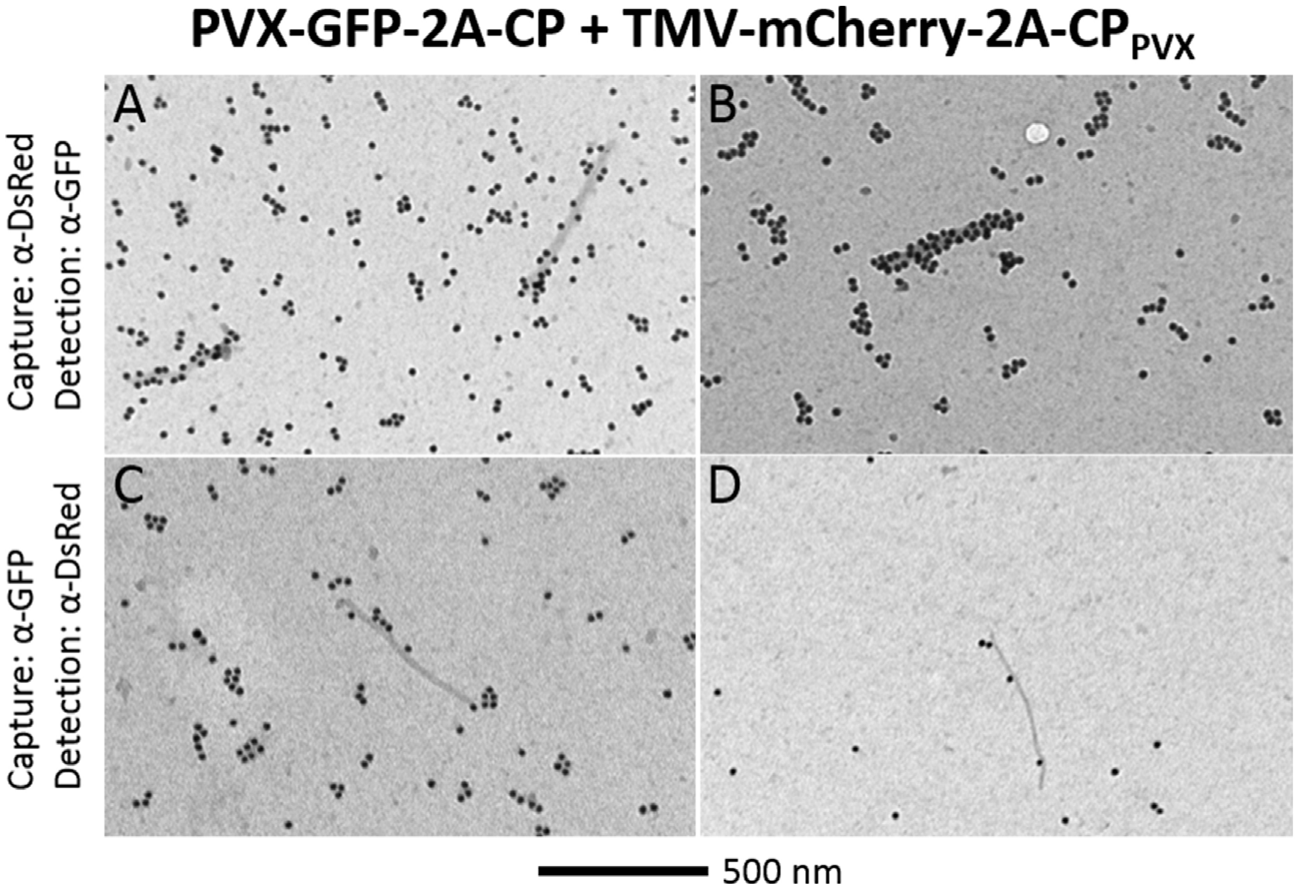
**TEM analysis of chimeric PVX particles displaying GFP and mCherry overcoat structures**. After coinfection with PVX-GFP-2A-CP and TMV-mCherry-2A-CP_PVX_, particles were captured with α-DsRed and decorated with α-GFP **(A,B)**, or captured with α-GFP and decorated with α-DsRed **(C,D)**. Bar = 500 nm.

The presence of both fluorescent proteins on the surface of PVX particles could be verified by capturing either with the DsRed antibody and labeling with α-GFP or vice versa. PVX particles could be captured from the preparations using either of the antibodies, confirming that at least one fluorescent protein can be found on the surface. Furthermore, some of these captured particles were decorated with the antibody against the second fluorescent protein, indicating that both fluorescent protein fusions can indeed assemble into one intact PVX particle (Figure 6).

### Coexpression of Split-mCherry-CP_PVX_ Fusions

We next used the BiFC system with split-mCherry to confirm the assembly of chimeric particles after coexpression of the different PVX CPs. Instead of mCherry and GFP, we used the N- and C-terminal portions of split-mCherry (N-mC or C-mC) as 2A-CP_PVX_ fusions, and expressed them using either a PVX vector or a TMV vector (Figure 1B). We combined the vectors pPVX-N-mC-2A-CP and pTMV-C-mC-2A-CP_PVX_ or pPVX-C-mC-2A-CP and pTMV-N-mC-2A-CP_PVX_ for coinfection studies. With this system, red fluorescence would only be generated if both parts of the split-mCherry CP fusions are expressed in the same cells and interact with each other.

**Figure 7:**
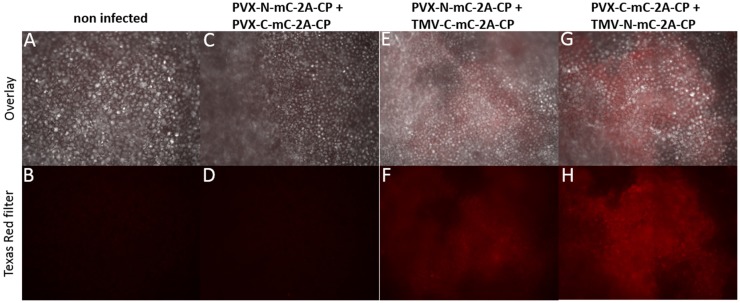
***Nicotiana benthamiana* cells analyzed for red fluorescence resulting from interactions of split-mCherry at 14 dpi**. Cells are shown in 10× magnification and specific excitation for mCherry was applied with a Texas Red filter **(B,D,F,H)**. The upper pictures show the overlay of transmitted light and the specific fluorescence excitation **(A,C,E,G)**. **(A,B)***N. benthamiana* non-infected, **(C,D)** plant infected with PVX-C-mC-2A-CP and PVX-N-mC-2A-CP, **(E,F)** coinfection of PVX-N-mC-2A-CP and TMV-C-mC-2A-CP_PVX_, and **(G,H)** coinfection of PVX-C-mC-2A-CP and TMV-N-mC-2A-CP_PVX_.

Plants were infected with each construct alone and pairwise combinations. No fluorescence was detected in single infections, or coinfections with two PVX- or TMV-based vectors expressing the different split-mCherry parts (Figures 7A–D and data not shown). Expression of the split-mCherry-2A-CP_PVX_ was confirmed by immunocapture RT-PCR and western blot analysis (Figures S3 and S5 in Supplementary Material). In plants coinfected with a combination of PVX and TMV vectors expressing different parts of the split-mCherry, only weak fluorescence was observed in infected leaves and was confirmed by fluorescence microscopy in the cells (Figures 7E–F). In all infections, the combination of the C-terminal part of the split-mCherry expressed from the PVX vector and the N-terminal part expressed from the TMV vector gave more intense red fluorescence than the reciprocal arrangement.

**Figure 8:**
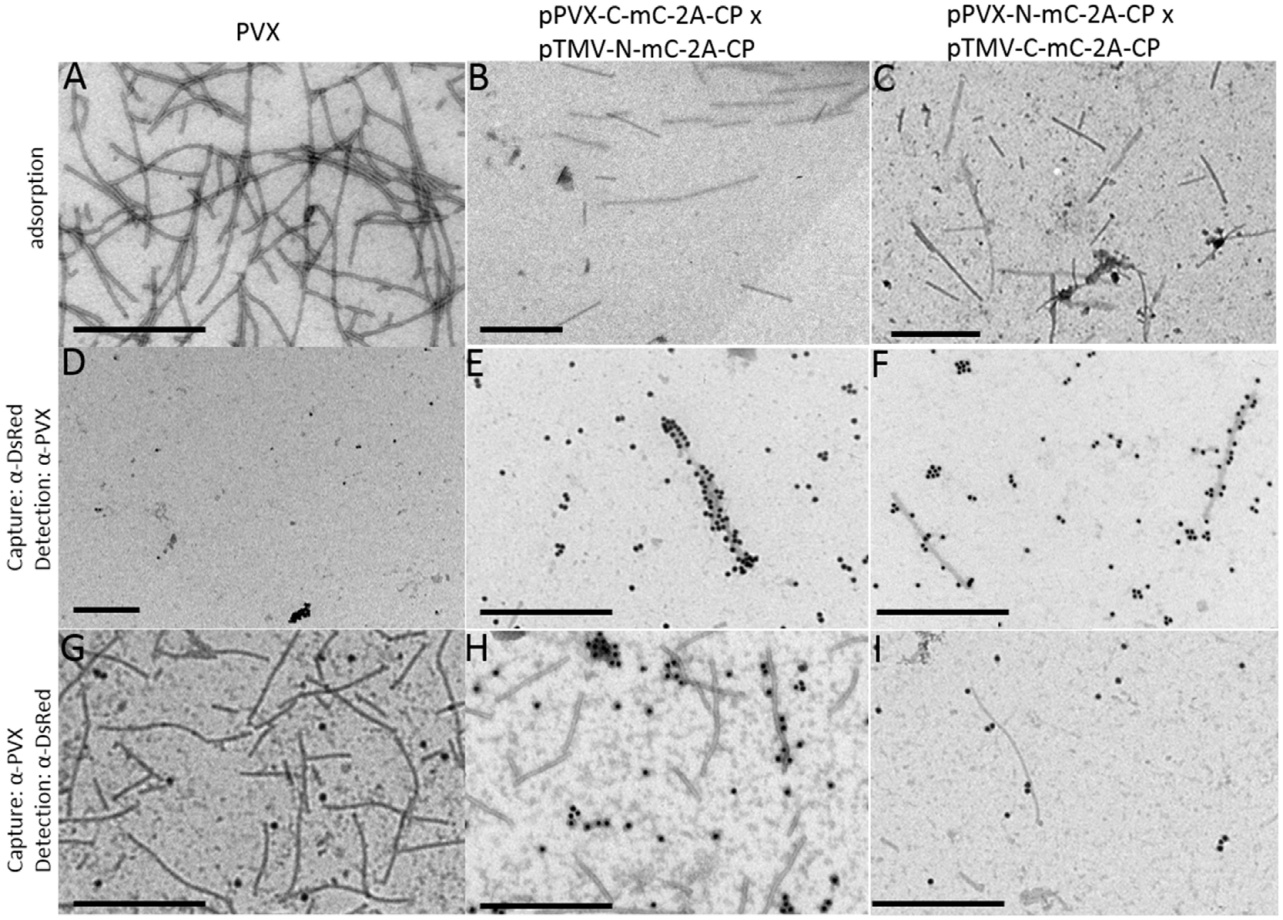
**Analysis of purified PVX particles in TEM studies with split-mCherry**. For TEM analysis of the particles, the preparations were either directly adsorbed to the grids **(A–C)** or captured with a DsRed-specific antibody and labeled with a PVX-specific antibody with GAR15nm **(D–F)** or captured with a PVX-specific antibody and labeled with a DsRed-specific antibody with GAR15nm **(G–I)**. PVX purification was used as control **(A,D,G)**, the purification of the coinfection of PVX-C-mC-2A-CP and TMV-N-mC-2A-CP_PVX_ **(B,E,H)**, and the coinfection of PVX-N-mC-2A-CP and TMV-C-mC-2A-CP_PVX_ **(C,F,I)** are displayed. Bars = 500 nm.

The split-mCherry data confirmed the coexpression of the two different viral vectors in a single cell, but we could not rule out the possibility that interactions might occur free in the cytosol, e.g., as CP dimers rather than in the context of an assembled particle. Therefore, particles were purified from infections with PVX-N-mC-2A-CP, PVX-C-mC-2A-CP and coinfections with a TMV vector expressing the other portion of split-mCherry, respectively. During purification via sucrose gradients, we obtained fractions (16–20) of the purified particles showing red fluorescence (Figure S4 in Supplementary Material). This indicated that the split-mCherry components may be able to form a functional fluorescent protein on the particle surface. We used TEM to determine whether the DsRed antibody could detect the split parts of mCherry even though the antibody could not detect the split-mCherry parts in western blot (Figure S5 in Supplementary Material). The adsorption grids showed again a mixture of TMV and PVX particles with an approximate ratio of 3:1 (Figures 8B,C). PVX particles were either captured with the DsRed antibody and labeled after capture with the PVX antibody or vice versa. We were unable to capture particles of the PVX control and particles displaying only one split-mCherry component by the DsRed antibody or label particles after capture with α-PVX when using the DsRed antibody. This confirmed that the DsRed antibody can only detect the complete mCherry protein and not the individual split components (Figure 8; Figure S5 in Supplementary Material). However, we were able to capture PVX particles from preparations derived from virus coinfections using the same α-DsRed antibody in both coexpressions of the split mCherry CP fusions. Furthermore, we were able to label the particles with the DsRed-specific antibody after capturing the particles with the PVX antibody from the PVX/TMV mixture (Figures 8H,I). We observed a better labeling with the α-DsRed antibody in particles derived from the coinfection if the C-terminal split-mCherry part is expressed by PVX. The detection of functional mCherry on the particle surface proves the assembly of hybrid chimeric PVX particles.

## Discussion

We have developed a novel production system for hybrid chimeric virus particles with at least two different polypeptides presented on the surface. With help of the FDMV 2A sequence, we were able to display rather large proteins on the recombinant particle (the fluorescent proteins GFP and mCherry), and also displayed the two split-mCherry portions, which interacted on the particle surface.

### Coexpression of Two CP_PVX_ Fusion Proteins Using PVX- and TMV-Based Vectors

We could show that a full-size PVX expression vector accepts the coexpression of additional PVX CP fusion protein delivered by a different viral expression vector. We found that PVX expression is enhanced in the presence of the TMV vector, resulting in brighter fluorescence for the PVX multiplication expressed fluorescent protein (Figure 3, mCherry fluorescence in Figure 3B compared to Figure 3C). The enhancement of PVX in a TMV-PVX coinfection is well known (Goodman and Ross, 1974) and was also observed in our system.

The fluorescence intensity of both proteins delivered by the TMV vectors was always lower than those delivered by the PVX vectors, although TMV accumulated to higher titers. This can possibly be explained by the distance of the fluorescent protein sequence relative to the 3′-end of the TMV genome (Culver et al., 1993). The distance between the genes for GFP or mCherry and the 3′-end is increased by the additional CP_PVX_ sequence, which limits the expression of the fluorescent protein. If the same fluorescent protein is expressed alone by the TMV vector, much more intense fluorescence and expression is observed (Lindbo, 2007). The influence of the relative distance of the gene of interest from the 3′-end of viral genomes could be explained by the impact of non-sense-mediated decay (NMD). In a recent study, it could be shown that long 3′-untranslated regions in subgenomic RNAs resulting from gene insertions into PVX vectors trigger the intrinsic restriction pathway in plants for plus-sense RNA viruses, namely NMD (Garcia et al., 2014). We speculate that this phenomenon might also explain why the genes located at the veriest 3′-end of viral genomes with 3′-coterminal subgenomic RNAs is in most cases expressed strongest. Thus, alternative expression strategies, e.g., 2A ribosomal skip sequences (Cruz et al., 1996; Gopinath et al., 2000), internal ribosome entry sites (Toth et al., 2001), or stop codon suppression (Hamamoto et al., 1993) could be advantageous. In PVX vectors, it can be observed that expression via a 2A-sequence fusion (this study and Cruz et al., 1996; Shukla et al., 2014) yield better results compared to an additional subgenomic promoter-like sequence (Baulcombe et al., 1995; Dickmeis et al., 2014).

Because coexpression of PVX CPs with different viral vectors was possible in the same cells, it was interesting to find out whether hybrid chimeric particles would be generated as well. We have identified PVX particles with different foreign PVX CP fusions assembled into the virion structure (Figure 6; Figure S2 in Supplementary Material), thus confirming the construction of chimeric PVX particles with a hybrid GFP and mCherry overcoat structure. This shows that PVX CP delivered by TMV can interact with PVX genomic RNA, giving initial hints that virion formation is not strictly coupled to CP production.

### Bimolecular Fluorescence Complementation

To analyze our expression system in more detail, we used the mCherry BiFC components (Fan et al., 2008) as fusion proteins in our PVX overcoat system. The BiFC system allows the analysis of protein interactions *in vivo* by fusing different parts of a fluorescent protein to the target proteins (Kodama and Wada, 2009). The fluorescent proteins are split and fluorescence can only be restored through the interaction of the target proteins. Plant viral CPs known to assemble without RNA were transiently expressed by *A. tumefaciens* for the evaluation of a monomeric red fluorescent protein (mRFP)-based BiFC system, because of their definite interaction capacity (Zilian and Maiss, 2011). The CPs of TMV, *Plum pox virus* (PPV), and *capsicum chlorosis virus* (CaCV) were shown to interact in the plant cells with itself after transient expression and by direct fusions of the split-mRFP portions to the CPs or a 7 amino acid linker sequence in between; however, full-sized particle formation has not been investigated. We used split-mCherry to confirm the coexpression of two viral vectors in the same cells. Restoration of the red fluorescence showed the coexpression of the two different PVX CP fusion proteins (Figure 7). Still, mCherry fluorescence does not necessarily confirm the interaction of the split-mCherry components during particle assembly, which may be explained by low potential interaction of free split-mCherry components (Morell et al., 2007) by the formation of CP dimers and oligomers.

We then analyzed the purified particles for the presence of mCherry restored from the split components. PVX particles displaying only one split-mCherry component were not labeled by the α-DsRed antibody, whereas some particles derived from the coexpression with the TMV/PVX combination were either captured or labeled by the DsRed antibody (Figure 8). Interestingly, we obtained a better labeling with the α-DsRed antibody if the C-terminal split-mCherry is expressed by the PVX vector, indicating a higher amount of particles with restored mCherry. The 9-kDa C-terminal split-mCherry is the smaller part of the mCherry protein (27 kDa). Thus, it seems to be more favorable for the construction of hybrid chimeric PVX particles to express the smaller peptide in the context of the PVX vector.

We have developed a plant expression system for the production of novel hybrid chimeric PVX particles, presenting two different overcoats on the surface. The PVX virion is a suitable epitope presentation system with 1270 CP subunits comprising each particle, thus a well-defined number of epitopes can be presented in high density to the immune system. PVX has been used successfully to present different epitopes and trigger an efficient immune response in animal models (Marusic et al., 2001; Lico et al., 2006). However, only single tandem repeats of epitopes have been presented on PVX as CP fusions (Uhde-Holzem et al., 2010). With our system, at least two different epitopes or even more in combinations with tandem repeats can be presented on the particle surface. We proved the system with fluorescent proteins, to directly visualize the expression of a second PVX CP *in planta* by another viral vector. We utilized the 2A sequence to obtain a hybrid overcoat structure on the PVX particles with the fluorescent proteins because these large proteins would impair particle assembly as a direct fusion. For epitope presentation, the epicoat structure with a particle completely decorated with epitopes would be more favorable.

During particle purification, TMV particles derived from the expression system were isolated as well (i.e., Figures 5 and 6). Thus, with a supplementary presentation of epitopes on the TMV particle surface, an even stronger vaccine candidate might be created. In all our particle purifications, we observed a ratio of TMV and PVX particles of ~3:1. The system, therefore, offers an additional possibility for tuning epitope presentation. Depending on the target pathogens, epitopes that induce a comparably low immune response can be presented on the TMV particles (which generate the largest number of purified particles in the mixture) and the comparably stronger epitopes can be displayed on PVX particles. The advantage of the coexpression is an easy purification of both rod-shaped particles in one procedure. Thus, our system provides a novel alternative expression platform for multicomponent/epitope immunization.

## Author Contributions

UC and CD provided the idea of the work and designed the experiments. CD conducted the experiments regarding the coexpression with the full-sized fluorescent proteins, whereas MH conducted the experiments with the split-mCherry constructs. UC, CD, MH, and RF participated in the interpretation of results and critically reviewed the manuscript. CD wrote the paper. All authors read and approved the final manuscript.

## Conflict of Interest Statement

The authors declare that the research was conducted in the absence of any commercial or financial relationships that could be construed as a potential conflict of interest.

## Abbreviations

BiFC, bimolecular fluorescence complementation; C-mC, C-terminal part of split-mCherry; CP, coat protein; FDMV, foot and mouth disease virus; GFP, green fluorescent protein; HCV, hepatitis virus C; ISEM, immunosorbent transmission electron microscopy; mRFP, monomeric red fluorescent protein; N-mC, N-terminal part of split-mCherry; NMD, non-sense-mediated decay.; ORF, open reading frame; PBS, phosphate-buffered saline; PVX, potato virus X; RdRp, RNA-dependent RNA polymerase; TEM, transmission electron microscopy; TGB, triple gene block; TMV, tobacco mosaic virus.
