# Supplementary material for: Production of Hybrid Chimeric PVX Particles Using a Combination of TMV and PVX-Based Expression Vectors
Source: Front Bioeng Biotechnol. 2015 Nov 20;3:189. doi: 10.3389/fbioe.2015.00189 (PMC4653303; doi:10.3389/fbioe.2015.00189)
Supplement: Supplementary file 1 [file Data_Sheet_1.DOCX]

# Supporting information:


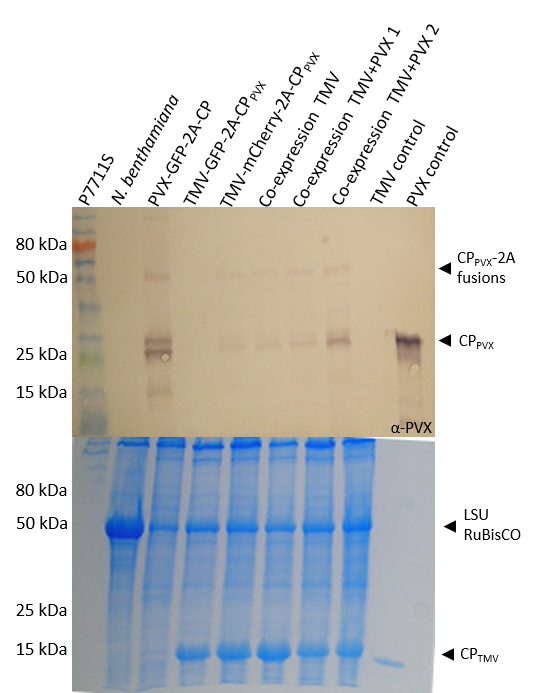


Figure S 1: Analysis of proteins extracted from *N. benthamiana* plants at 12 dpi expressing 2A-CP_PVX_ fluorescent protein fusions following co-infection with PVX and TMV vectors. The western blot (upper panel) shows the detection of the PVX CP with a α-PVX and GAR^AP^. The lower panel shows the polyacrylamide gel stained with Coomassie Brilliant Blue after blotting. Plant sap was sampled from single infections (PVX-GFP-2A-CP, TMV-GFP-2A-CP_PVX_ or TMV-mCherry-2A-CP_PVX_) or from co-infections with two TMV-based vectors (co-expression TMV) or TMV/PVX combinations. P7711S: protein ladder (NEB), *N. benth*: non-infected plant, co-expression TMV + PVX plant 1: TMV-GFP-2A-CP_PVX_ and PVX-mCherry-2A-CP, co-expression TMV + PVX plant 2: TMV-mCherry-2A-CP_PVX_ and PVX-GFP-2A-CP, TMV control: purified TMV particles (0.5 µg), PVX control: purified PVX 201 particles (0.5 µg). The arrowheads show the positions of the CP_PVX_-2A fusions and CP_PVX_ in the western blot and CP_TMV_ as well as the large subunit of RuBisCO (LSU RuBisCO) in the Coomassie-stained gel.


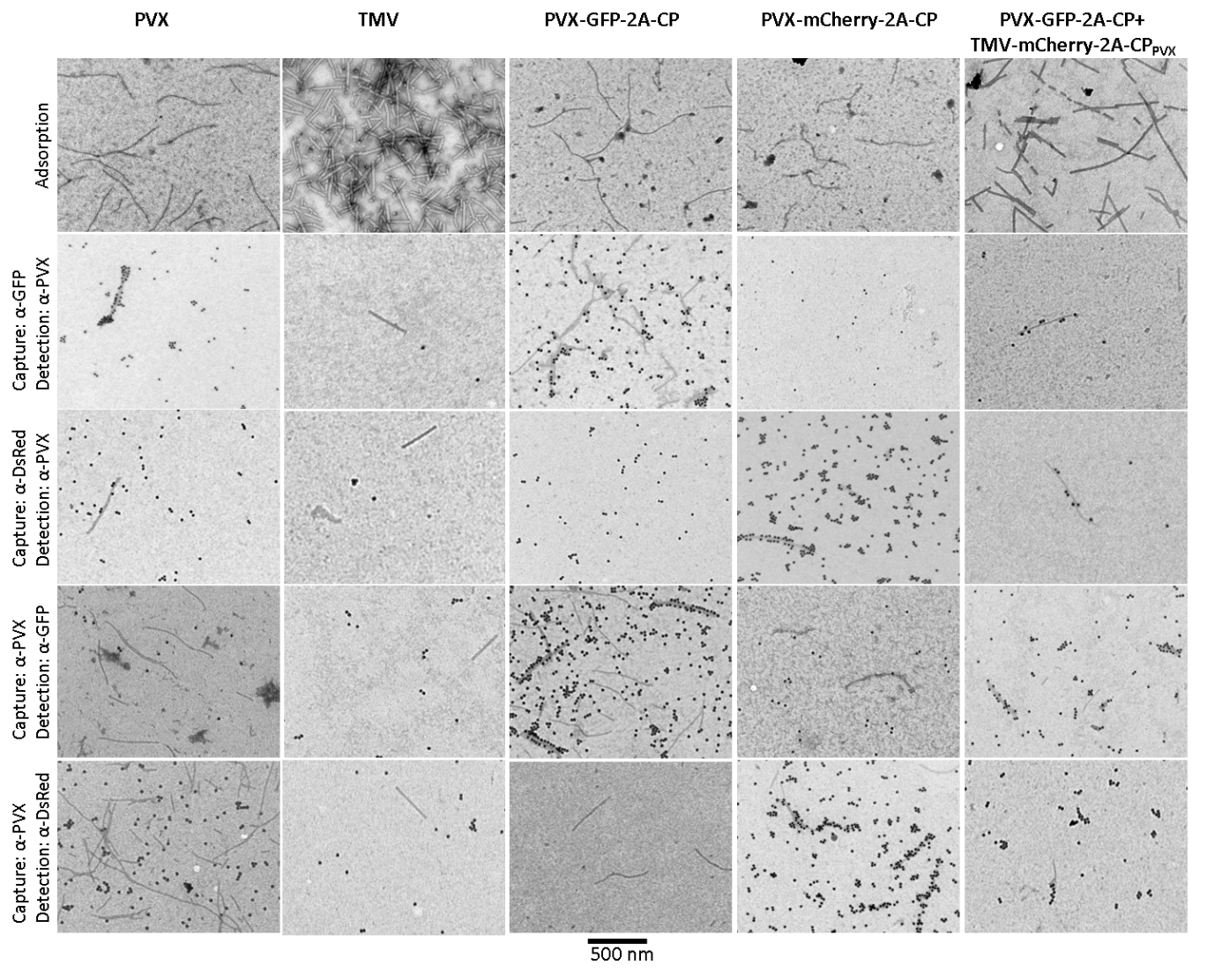


Figure S2: TEM analysis of particle purifications captured and labeled with specific antibodies. The particles were either directly adsorbed to the grids, captured with α-GFP or α-DsRed and decorated with α-PVX, or captured with α-PVX and decorated with α-GFP or α-DsRed. To confirm the specific binding of the antibodies on the particles, PVX, TMV, PVX-GFP-2A-CP and PVX-mCherry-2A-CP purifications were used as controls. Furthermore the co-expression of PVX overcoat structures by co-infection of PVX-GFP-2A-CP and TMV-mCherry-2A-CP_PVX_ was analyzed. Bar = 500 nm


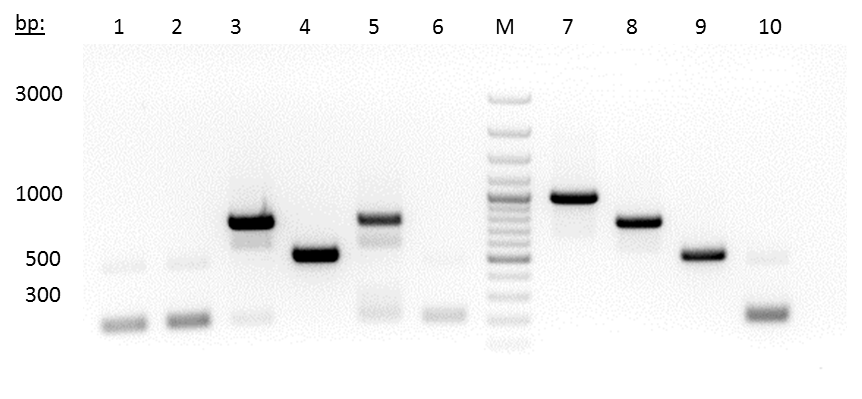


Figure S 3: Immunocapture and RT-PCR analysis of plant sap from N. benthamiana expressing N- or C-terminal split-mCherry halves as 2A-CP_PVX_ fusions. 1: PBS in immunocapture, 2: non-infected plant material, 3: PVX-N-mC-2A-CP, 4: PVX-C-mC-2A-CP, 5 and 6: different plants infected with PVX-N-mC-2A-CP +TMV-C-mC-2A-CP, M: DNA ladder 100 bp+ (fermentas), 7: DNA control PVX-mCherry-2A-CP, 8: DNA control PVX-N-mC-2A-CP, 9: DNA control PVX-C-mC-2A-CP, 10: negative control no template.


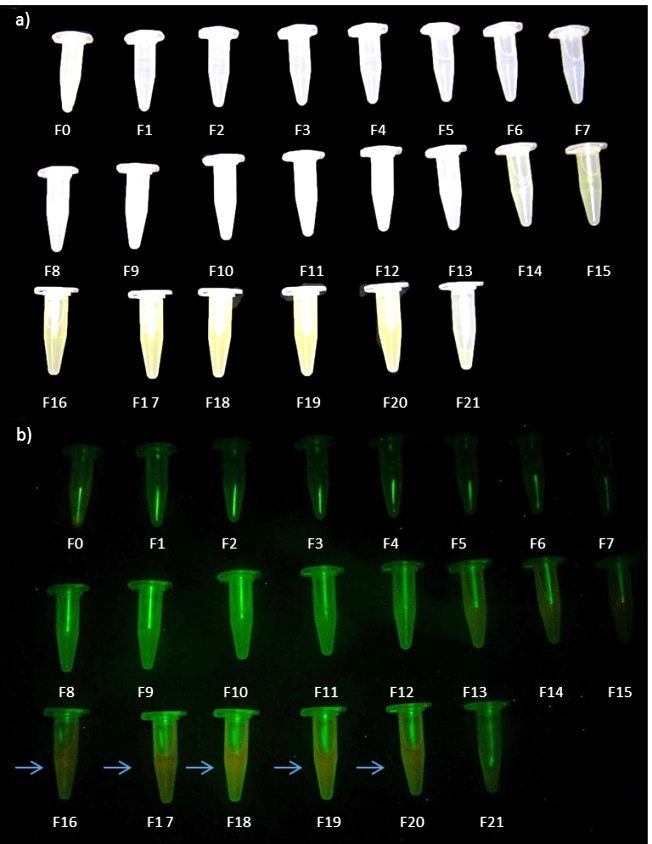


Figure S4: Reaction tubes with the sampled fractions of the particle purification of the split-mCherry-2A-CP fusions under normal light (a) and with excitation of mCherry (b). After centrifugation of the particles mixture on a sucrose gradient, 1.5ml-fractions were collected. The fractions F16 to F20 showed a red fluorescence after excitation with green light.


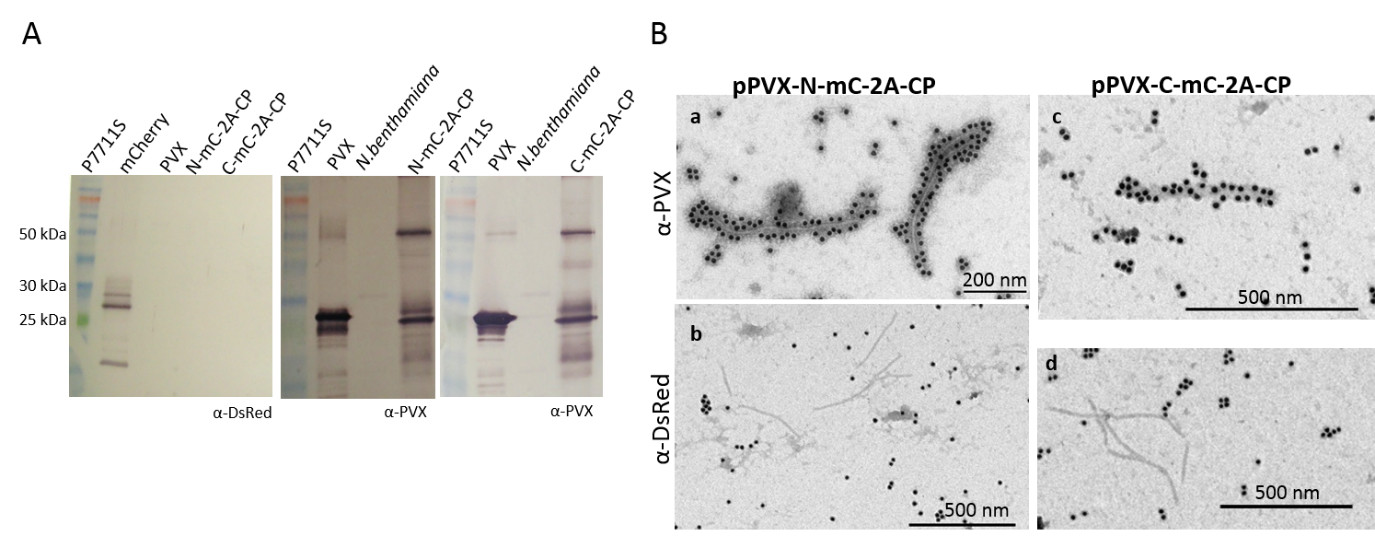


Figure S5: Confirmation of the specific binding of the DsRed antibody to only full-sized mCherry by western blot. A) Purified particles or proteins were applied on PAA gels and incubated with the α-DsRed and α –PVX antibody and GAR^AP^. P7711S: protein ladder (NEB), mCherry: purified mCherry-his6 control (1 µg), PVX: PVX control (1 µg), N-mC-2A-CP: PVX-N-mCherry-2A-CP purified particles (1 µg), C-mC-2A-CP: PVX-C-mCherry-2A-CP purified particles (1 µg), *N. benthamiana*: non infectedplant extract. B) TEM pictures of adsorption grid with either PVX-N-mC-2A-CP labeled by α-PVX (a) or α-DsRed (b), or PVX-C-mC-2A-CP labeled by α-PVX (c) or α-DsRed (d).
